# Atorvastatin inhibits osteoclastogenesis by decreasing the expression of RANKL in the synoviocytes of rheumatoid arthritis

**DOI:** 10.1186/ar4018

**Published:** 2012-08-17

**Authors:** Jeong Yeon Kim, Eun Young Lee, Eun Bong Lee, Yun Jong Lee, Hyun Jung Yoo, Jiyong Choi, Yeong Wook Song

**Affiliations:** 1 Division of Rheumatology, Department of Internal Medicine, Seoul National University Hospital, 101 Daehak-ro, Jongno-gu, Seoul, 110-744, Korea; 2 Division of Rheumatology, Department of Internal Medicine, Seoul National University Bundang Hospital, 82, Gumi-ro 173 Beon-gil, Bundang-gu, Seongnam-si, Gyeonggi-do, 463-707, Korea; 3 WCU Department of Molecular Medicine and Biopharmaceutical Sciences, Medical Research Institute, Seoul National University College of Medicine, 103 Daehak-ro, Jongno-gu, Seoul, 110-799, Korea

## Abstract

**Introduction:**

Statins, hydroxymethylglutaryl-coenzyme A reductase inhibitors, have been reported to have antiinflammatory and/or immunomodulatory effects and prophylactic and therapeutic effects in collagen-induced arthritis, an experimental model of rheumatoid arthritis (RA). The authors undertook to determine the effect of atorvastatin on the expressions of osteoprotegerin (OPG) and receptor activator of nuclear factor κB ligand (RANKL) in RA fibroblast-like synoviocytes (FLSs), to identify the mechanisms responsible for these effects, and to determine whether the statin inhibits osteoclastogenesis.

**Methods:**

FLSs isolated from five RA patients were cultured in the presence of 20 ng/ml of tumor necrosis factor-α (TNF-α) with or without atorvastatin. RANKL expressions were assayed with Western blotting and enzyme-linked immunosorbent assay. RANKL, RANK, and OPG expression were assayed with reverse transcription-polymerase chain reaction (RT-PCR). Osteoclast formation was assayed by counting cells after staining for tartrate-resistant acid phosphatase in cocultures of peripheral blood mononuclear cells (PBMCs) and RA FLSs.

**Results:**

Atorvastatin inhibited the expression of RANKL in RA FLSs in a dose-dependent manner, and the suppression of RANKL was prevented by mevalonate. However, OPG expression was not affected by atorvastatin in RA FLSs, and atorvastatin did not affect RANK expression in CD14^+ ^cells. Conversely, atorvastatin suppressed TNF-α-induced p38 phosphorylation in RA FLSs and significantly reduced TRAP-positive multinucleated osteoclast formation in the coculture of PBMCs and RA FLSs.

**Conclusion:**

These results suggest that atorvastatin inhibits osteoclastogenesis and bone destruction in RA patients.

## Introduction

Receptor activator of nuclear factor κB ligand (RANKL), and its receptor, RANK, have been found to be key factors in the stimulation of osteoclast formation, and they have also been suggested to play major roles in inflammation-induced bone loss and joint destruction in arthritis [1,2]. The soluble tumor necrosis factor (TNF)-receptor molecule, osteoprotegerin (OPG), is a natural inhibitor of RANKL. OPG binds to RANKL and prevents it from interacting with RANK, and thus, the balance between RANKL and OPG in the bone microenvironment regulates bone resorption [3].

Rheumatoid arthritis (RA) is characterized by inflammatory synovitis and progressive destruction of joint cartilage and bone [4,5]. Furthermore, RA patients exhibit high serum levels of OPG and soluble RANKL [6]; RANKL mRNA is present in the synovial lining layer in RA [7]. However, RANKL is not expressed in normal synovium, which suggests a link between RANKL expression and the development of synovial lesions in RA [8]. In addition, recent studies provided genetic evidence that RANKL and osteoclasts are central players in the inflammatory destruction of bone [9] and that enhanced RANKL expression in synoviocytes induced by synovial inflammation may be critical for osteoclastogenesis [10].

Statins, hydroxymethylglutaryl-coenzyme A (HMG-CoA) reductase inhibitors, constitute a family of chemically related molecules with lipid-lowering effects. Statins are extensively used in medical practice, and large-scale clinical trials have demonstrated their efficacies at reducing cardiovascular-related morbidities and mortalities [11,12]. Furthermore, increasing clinical and experimental evidence indicates that statins might have general antiinflammatory and immunomodulatory effects; research studies conducted over the last 10 years have elucidated a number of mechanisms by which statins may exert antiinflammatory effects [13,14]. More recently, the beneficial effects of statins have been extended to the direct immunomodulation of monocyte-mediated inflammatory processes (including chronic inflammatory diseases, such as atherogenesis and RA), independent of their effects on cholesterol levels [15-17].

Atorvastatin has been shown to have antiinflammatory potential in RA clinical trials [18,19]. However, the effects of atorvastatin on human osteoclasts have not been determined. In this study, we examined the effects of atorvastatin on the expressions of OPG and RANKL in fibroblast-like synoviocytes (FLSs) from RA patients and the mechanisms involved, and in addition, we sought to determine whether the statin inhibits osteoclastogenesis.

## Materials and methods

### Chemicals

Atorvastatin (Pfizer, New York, NY, USA) was prepared as a suspension in dimethyl sulfoxide (DMSO; Sigma, St. Louis, MO, USA). Mevalonate (Sigma) was dissolved in 1 N NaOH (pH 7.1). SB2035820, p38 inhibitor, was purchased from Cell Signaling Technology (Danvers, MA, USA).

### Primary culture of FLS

Synovial tissues were obtained from five patients undergoing joint-replacement surgery. All five patients fulfilled the 2010 rheumatoid arthritis classification criteria of RA by the American College of Rheumatology/European League Against Rheumatism collaborative initiative [20]. This study was approved by the Institutional Review Board, and informed consent was obtained from all patients. Their clinical characteristics are shown in Table 1.

**Table 1:** Clinical characteristics of the patients with rheumatoid arthritis

Patient	Disease duration (months)	Site of surgery	Gender	Medication
**1**	180	Knee	Female	Prednisolone, 5 mg/d; celecoxib, 200 mg/d; gasmotine, 2.5 mg/d
**2**	36	Knee	Female	Cyclosporin A, 100 mg/d; triamcinolone, 2 mg/d
**3**	240	Knee	Male	Prednisolone, 5 mg/d; hydroxychloroquine, 200 mg/d; sulfasalazine, 500 mg/d
**4**	180	Hip	Female	Hydroxychloroquine, 200 mg/d
**5**	187	Knee	Female	Deflazacort, 5 mg/d; methotrexate, 12.5 mg/wk; leflunomide, 10 mg/d; hydroxychloroquine, 200 mg/d

Synovial tissues were washed with phosphate-buffered saline (PBS), minced, and digested for 2 hours at 37°C in Dulbecco Modified Eagle's Medium-High Glucose (DMEM-HG; JBI, Daejeon, Korea) containing 1 mg/ml type II collagenase (Worthington Biochemical Corporation, NJ, USA). The digested tissues were filtered through a 70-μm cell strainer (Becton Dickinson, Franklin Lakes, NJ, USA). Cell suspensions were centrifuged at 1,400 rpm for 20 minutes at room temperature, and cell pellets were resuspended in DMEM-HG containing 1% penicillin-streptomycin (Gibco/BRL, Grand Island, NY, USA) and 10% heat-inactivated fetal bovine serum (FBS; Gibco/BRL). The cells were then plated in 100-mm culture dishes (Becton Dickinson) and incubated in a humidified 5% CO_2 _atmosphere. On reaching confluence, cells were detached with 0.1% trypsin-EDTA (Gibco/BRL) and split into 5 × 10^5 ^cells per dish. For all experiments, three- to five-passage synovial fibroblasts were used.

### Immunomagnetic selection of CD14^+ ^cells

CD14^+ ^cells were purified by MACS according to the manufacturer's instructions (Miltenyi Biotech, Auburn, CA, USA). In brief, 10^7 ^PBMCs were resuspended in 80 μl of cold PBS containing 0.5% BSA and 2 m*M *EDTA (MACS buffer). Microbeads (20 μl) conjugated with mouse anti-human CD14IgG2a (Miltenyi Biotech) were then added to cells and incubated for 15 minutes at 4°C. Cells were then washed and resuspended in 3 ml of MACS buffer and then applied to a preequilibrated sterile LS separation column (Miltenyi Biotech) placed in a MidiMACS magnet. The column was then washed 3 times with MACS buffer, and CD14^+ ^cells were eluted with 5 ml of MACS buffer after removing the column from the magnet.

### Methylthiazol tetrazolium assay

FLSs or PBMCs were cultured to confluence in 96-well plates (Becton Dickinson) and treated with M-CSF (2 ng/ml), 1,25-dihydroxyvitamin D_3 _(10^-7 ^*M*), atorvastatin for 24 hours (10 to 100 μ*M*) or 3 weeks (0.001 to 0.1 μ*M*) in a 5% CO_2 _incubator. Methylthiazol tetrazolium (MTT) labeling reagent (500 μg/ml; Sigma) was added to FLSs or PBMCs in wells and incubated for 2 hours at 37°C. Isopropanol supplemented with 0.4 *M *HCl was then added to release formazan dye from cells. Formazan absorbance in solution was measured by using a microtiter-plate enzyme-linked immunosorbent assay (ELISA) reader at 570 nm.

### Propidium iodide staining

FLSs were cultured in 100-mm culture dishes and treated with TNF-α (20 ng/ml) and atorvastatin (10 to 100 μ*M*) for 24 hours. Cells were harvested by using 0.1% trypsin-EDTA, washed twice in PBS containing 0.1% (wt/vol) NaN_3_, and then 1 ml of cold 70% (vol/vol) ethanol was added to cell pellets. Cells were then vortexed and incubated at 4°C for 1 hour, washed twice, and resuspended in 0.1 ml of PBS containing 0.1% NaN_3_. For DNA staining, cells were treated with 1 mg/ml of RNase A (Sigma), resuspended in 0.5 ml of PBS containing 50 mg/ml propidium iodide (PI; Sigma), and incubated for 30 minutes at 4°C in the dark. They were then analyzed with flow cytometry (FACS Caliber, BD Science), and the sub-G_0_/G_1 _portion (the M1 fraction) was considered to be the apoptotic fraction.

### Reverse transcription-polymerase chain reaction and real-time real-time PCR

Total RNA was extracted from FLSs or CD14^+ ^cells by using TRIzol reagent (Invitrogen Life Technologies, Carlsbad, CA, USA) according to the manufacturer's instructions. In brief, FLSs or CD14^+ ^cells were cultured in six-well plates in the presence or absence of TNF-α (20 ng/ml), atorvastatin for FLSs (10 to 100 μ*M*), for CD14^+ ^cells (0.01 to 1 μ*M*) for 24 to 72 hours. The cells were then lysed by adding 1 ml TRIzol reagent, and lysates were collected in 1.5-ml microtubes, to which was added 0.2 ml of chloroform (Sigma). The microtubes were then centrifuged at 12,000 *g *for 15 minutes at 4°C, and supernatants were transferred to new microtubes. Isopropyl alcohol (0.5 ml) was then added to precipitate RNA, and microtubes were centrifuged at 12,000 *g *for 8 minutes at 4°C. Pellets were washed with 75% ethanol, dried at room temperature, and resuspended in 10 to 20 μl nuclease-free water (Applied Biosystems/Ambion, Austin, TX, USA). RNA concentrations were determined by measuring absorbance at 260 nm.

First-strand cDNA was synthesized by using a Power cDNA Synthesis Kit (Intron Biotechnology, Seongnam, South Korea). The reaction was conducted in 20 μl of buffer containing 1 μg of total RNA, 0.2 m*M *oligo (dT)_15 _primer, 5× RT buffer, 40 m*M *DTT, 10 units of RNase inhibitor, 2.5 m*M *deoxynucleotide triphosphate (dNTP) mixture, and 5 units of AMV reverse transcriptase. After incubation at 37°C for 1 hour, the reaction was stopped by heating at 70°C for 15 minutes. To remove the remaining RNA, 1 μl of *Escherichia coli *RNase H (4 mg/ml) was added to reaction mixtures and this was followed by incubation at 37°C for 30 minutes. The housekeeping gene, glyceraldehyde 3-phosphate dehydrogenase (GAPDH), was used as an internal control for determining gene expression. The primers used to detect RANKL (S, 5' GCC AGT GGG AGA TGT TAG 3'; AS, 5' TTA GCT GCA AGT TTT CCC 3'); RANK (S, 5' TTA AGC CAG TGC TTC ACG GG 3'; AS, 5' ACG TAG ACC ACG ATG ATG TCG C 3'); OPG (S, 5' TGC TGT TCC TAC AAA GTT TAC C 3'; AS, 5' CTT TGA GTG CTT TAG TGC GTG 3'), and GAPDH (S, 5' GCT CTC CAG AAC ATC ATC CC 3'; AS, 5' CGT TGT CAT ACC AGG AAA TG 3').

PCR amplification of cDNA was performed in an automated thermal cycler (GeneAmp PCR System 2400, Applied Biosystems) in a final volume of 20 μl containing 1 to 4 μl of cDNA, 20 m*M *Tris-HCl (pH 8.4), 50 m*M *KCl, 1.5 m*M *MgCl_2_, 0.1% Triton X-100, 0.2 m*M *dNTP mixture, 0.5 pmol of each primer, and 5 units of *Taq *DNA polymerase (Promega). After PCR, the amplified products were analyzed with electrophoresis in 1.5% agarose gel and visualized with ethidium bromide staining under UV illumination. Band intensities of PCR products were measured by using a UV/VIS Vilber Lourmat digital camera and the software BioCaptMW (Vilber Lourmat, Marne-la-Vallée, France).

Real-time PCR was performed with the ABI 7500 real-time PCR system (Applied Biosystems). Total RNA was extracted by using an RNeasy kit (Qiagen, Valencia, CA, USA). Synthesis of cDNA was performed by using an SuperScript III First-Strand Synthesis System (Invitrogen) according to the manufacturer's protocol. Quantitative real-time PCR was performed by running a QuantiTect SYBR Green PCR Kit (Qiagen). Primer sequences were as follows; GAPDH, forward: CAATGACCCCTTCATTGACC; GAPDH, reverse: TGGACTCCACGACGTACTCA; and RANKL, forward GCTTGAAGCTCAGCCTTTTG:; and RANKL, reverse: CGAAAGCAAATGTTGGCATA. The reactions were incubated at 94°C for 15 minutes for one cycle, and then at 94°C (15 seconds), 59°C (30 seconds), and 72°C (30 seconds) for 40 cycles. The quantity of mRNA was calculated by using the threshold cycle (C_t_) value for amplification of human RANKL and for human GAPDH as a reference gene. Relative gene expression was determined by the 2^ΔΔCt ^method.

### Western blot analysis

Protein expressions were measured with Western blotting. In brief, cells were washed twice with ice-cold PBS, scraped into microfuge tubes, pelleted by centrifugation, and suspended in PRO-PREP protein-extraction buffer (Intron). After 30 minutes of incubation on ice, samples were centrifuged at 14,000 rpm for 30 minutes at 4°C. Protein concentrations were measured by using Bio-Rad Protein Assay reagent (Bio-Rad) with bovine serum albumin as a standard. Sodium dodecylsulfate-polyacrylamide gel electrophoresis (SDS-PAGE) was carried out by using a mini-protein system (Bio-Rad). Dissolved protein samples were combined with sample buffer (125 m*M *Tris (pH 6.8), 5% glycerol, 2% SDS, 1% 2-mercaptoethanol, and 0.006% bromophenol blue) boiled for 5 minutes, and immediately cooled. Equivalent amounts of protein samples (50 μg/lane) were loaded onto 12% SDS gels. Electrophoresis was carried out in a running buffer at 100 V for 2 hours. Proteins were transferred from gels onto polyvinylidene difluoride (PVDF) membranes (Millipore, Bedford, MA, USA; 90 minutes at 30 V) in transfer buffer (192 m*M *glycine, 25 m*M *Tris-HCl, pH 8.3, 0.02% SDS, and 20% vol/vol TBS-Tween buffer (20 m*M *Tris, pH 8.0, 150 m*M *NaCl, and 0.1% Tween 20)) for 1 hour at 4°C. After blocking with 5% nonfat dried milk in TBS-Tween buffer for 1 hour, rabbit polyclonal antibody to human RANKL (R&D), rabbit monoclonal anti-Akt/phospho-Akt, anti-ERK/phospho-ERK, anti-JNK/phospho-JNK, anti-p38/phospho-p38 antibodies (all from Cell Signaling Technology), and rabbit monoclonal anti-actin antibody (Sigma) were added and incubated for 12 hours. Membranes were then washed and incubated with HRP-conjugated goat anti-rabbit IgG polyclonal antibodies (1:1,000; Jackson Immunoresearch, Philadelphia, PA, USA). Chemiluminescent substrate (Amersham Life Science, Arlington Heights, IL, USA) was added, and incubation was continued for 10 minutes. Blots were then exposed to radiographic film.

### Enzyme-linked immunosorbent assay

The secretion of sRANKL was detected by using RANKL ELISA kit (Peprotech), according to the manufacturer's directions.

### Coculture system for osteoclastogenesis

Blood was collected from healthy volunteers, and PBMCs were isolated by centrifugation over Ficoll/Paque at 1,700 rpm for 30 minutes. PBMCs were resuspended in α-MEM containing 10% FBS and 2 ng/ml M-CSF (Sigma) and seeded at 2 × 10^5 ^cells/well in 96-well culture plates. On the following day, FLSs were added at 2 × 10^4 ^cells/well to adherent PBMCs and cocultured for 3 weeks in α-MEM containing 10% FBS, 100 IU/ml benzyl penicillin, 100 mg/ml streptomycin, 2 ng/ml M-CSF, and 10^-7 ^*M *1,25-dihydroxyvitamin D_3_. The culture medium was replaced every 3 days. Adherent cells were stained for tartrate-resistant acid phosphatase (TRAP) by using a commercially available kit (Sigma), as previously described (10). TRAP-positive multinucleated cells containing three or more nuclei were identified as osteoclasts and counted with light microscopy. All experiments were carried out 3 times in triplicate.

### Statistical analysis

Data are presented as mean ± SD. Statistical analysis was performed by using SPSS version 12 (SPSS Inc., Chicago, IL, USA), and *P *values of < 0.05 were considered significant.

## Results

### Effects of atorvastatin on RANKL in the FLSs of RA patients

**Figure 1:**
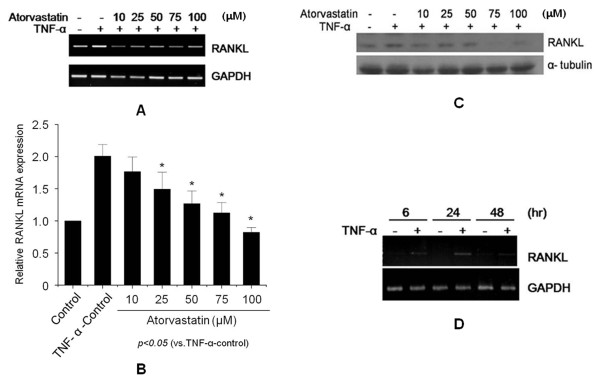
**Effects of atorvastatin on receptor activator of nuclear factor κB ligand (RANKL) expression in fibroblast-like synoviocytes (FLSs) from rheumatoid arthritis (RA) patients**. FLSs were isolated from three RA patients (patients numbers 1, 2, and 3) and cultured in the presence of tumor necrosis factor (TNF)-α (20 ng/ml) with or without atorvastatin for 24 hours. RANKL expressions were analyzed with RT-PCR **(A)**, real-time PCR **(B)**, and Western blotting **(C). (D) **TNF-α induced expression of RANKL mRNA in the FLSs of RA patients. Total RNA was extracted from FLSs cultured in the absence (-) or in the presence (+) of TNF-α (20 ng/ml) for indicated times. Total RNA was analyzed with RT-PCR. Results are representative of three independent experiments.

**Figure 2:**
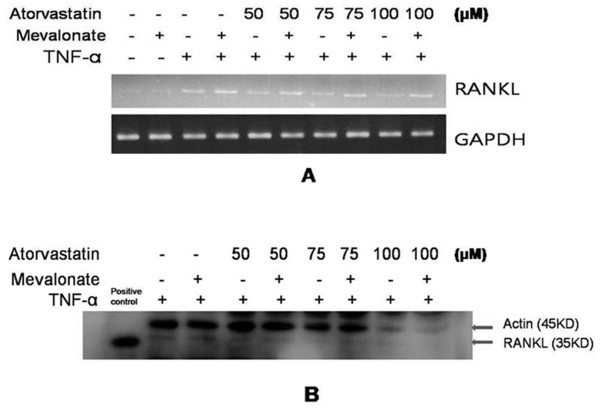
**Effects of mevalonate on the statin-induced suppression of RANKL**. FLSs from RA patients (patients number 1 through 3) were cultured in the presence of TNF-α (20 ng/ml), and then atorvastatin (50 to 100 μ*M*) and mevalonate (100 μ*M*) were added for 24 hours. RANKL expressions were analyzed with RT-PCR **(A) **and Western blotting **(B)**. A representative experiment from three independent experiments is shown.

FLSs were cultured for 24 hours with atorvastatin in the presence of TNF-α (20 ng/ml), and assayed for RANKL expression with RT-PCR and real-time PCR. Atorvastatin significantly and dose-dependently inhibited the expression of RANKL mRNA in the FLSs of RA patients (*P *< 0.05) (Figure 1A, B). Western blotting of these cells showed that atorvastatin also dose-dependently suppressed RANKL protein levels (Figure 1C). Simvastatin has been reported to reduce arthritis incidence, activity, and histologic scores in a collagen-induced arthritis model, and simvastatin has been shown to have antiinflammatory potential in RA patients (18,22). Therefore, we determined whether simvastatin inhibits the expression of RANKL. Simvastatin also significantly and dose-dependently inhibited the expression of RANKL mRNA and protein in the FLSs of RA patients (see Additional file 1, Figure S1). To determine the most effective time for TNF-α stimulation, RANKL expression was analyzed with RT-PCR after treating the FLSs of RA patients for 24 to 48 hours with TNF-α (20 ng/ml). RANKL mRNA expression was found to peak after 24 hours of stimulation with TNF-α (20 ng/ml) (Figure 1D). Because mevalonate is synthesized from 3-hydroxy-3-methylglutaryl coenzyme A (HMG-CoA) by HMG-CoA reductase, HMG-CoA reductase inhibitors, like statins, reduce the entry of mevalonate into the cholesterol synthesis pathway. To determine whether mevalonate prevents the inhibition of RANKL expression by atorvastatin, mevalonate (100 μ*M*) was cotreated with atorvastatin. RT-PCR and Western blotting showed that mevalonate prevented the suppression of RANKL expression by atorvastatin (Figure 2). Also, mevalonate prevented the inhibition of RANKL expression by simvastatin (see Additional file 2, Figure S2).

### The effects of atorvastatin on cell viability and apoptosis

**Figure 3:**
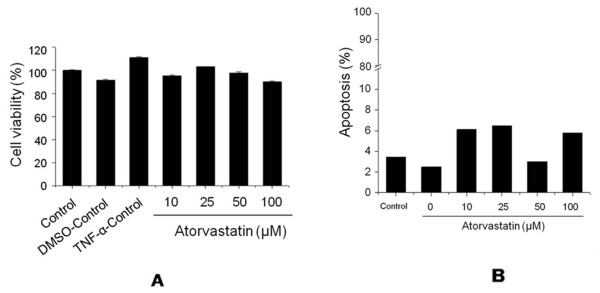
**The effects of atorvastatin on cell viability and apoptosis**. **(A) **Effects of atorvastatin on the viability of FLSs from an RA patient (patients 1, 2, and 3). Cells were cultured for 24 hours in the presence of TNF-α (20 ng/ml) and atorvastatin at different concentrations (10 to 100 μ*M*). Cell viabilities were determined by using MTT assays. **(B) **Effects of atorvastatin on the apoptosis of FLSs as determined by propidium iodide staining.

The effect of atorvastatin on cell viability was determined by using an MTT-based assay (Figure 3A). Apoptosis was measured by staining with propidium iodide (Figure 3B). Neither cell viability nor apoptosis was affected by atorvastatin at concentrations of 10 to 100 μ*M*. Simvastatin did not affect cell viability and apoptosis (see Additional file 3, Figure S3).

### Effects of atorvastatin on OPG in the FLSs of RA patients

**Figure 4:**
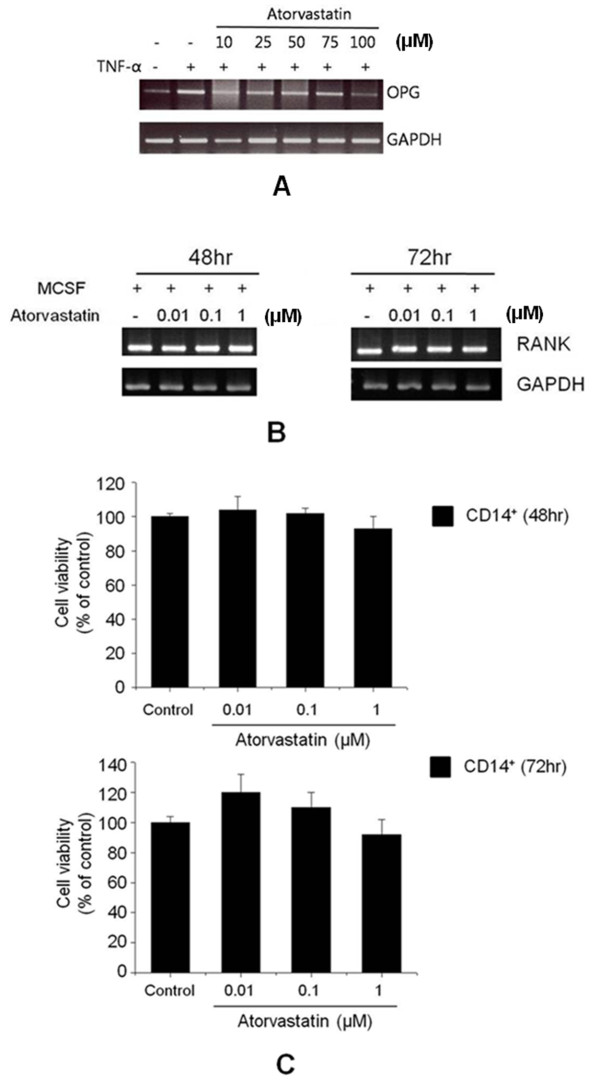
**Effects of atorvastatin on osteoprotegerin (OPG) expression in FLSs and RANK expression in CD14^+ ^cells of RA patients (patients 1, 4, and 5)**. **(A) **RT-PCR was used to analyze OPG mRNA expression in FLSs cultured for 24 hours with 20 ng/ml TNF-α and atorvastatin (10 to 100 μ*M*). The results shown are representative of three independent experiments. **(B) **CD14^+ ^cells were cultured for 48 or 72 hours in the presence of 2 ng/ml of macrophage colony-stimulating factor and different concentrations of atorvastatin (0.01 to 1 μ*M*). RANK mRNA expression was not affected by atorvastatin according to RT-PCR results. **(C) **Atorvastatin had no effect on the viability of CD14^+ ^cells at concentrations up to 1 μ*M*. The results are representative of three independent experiments.

The expressions of OPG mRNA were examined in FLSs from patients cultured for 24 hours with RT-PCR. Atorvastatin did not affect OPG expression in these cells (Figure 4A). We also assayed the secretion of OPG into the conditioned media of FLSs cultured in the presence of atorvastatin, and the statin was found to affect OPG secretion (data not shown).

### Effect of atorvastatin on RANK in CD14^+ ^cells

The effect of atorvastatin on RANK in osteoclast precursor cells was evaluated by purifying CD14^+ ^cells from total PBMCs by using a magnetic cell-separation (MACS) system. Atorvastatin did not affect RANK mRNA expression in CD14^+ ^cells (Figure 4B), and the viabilities of CD14^+ ^cells were not affected by atorvastatin at concentration up to 1 μ*M *(Figure 4C).

### Effect of atorvastatin on tumor necrosis factor-α-induced signal-transduction pathways in FLSs from rheumatoid arthritis patients

**Figure 5:**
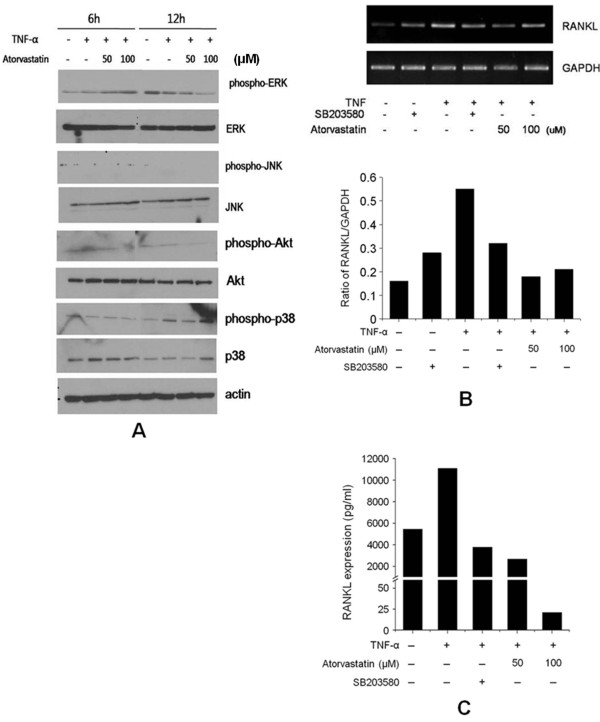
**The effect of atorvastatin on the phosphorylations of ERK, JNK, Akt, and p38 MARK, induced by TNF-α in FLSs from RA patients (patients 1, 4, and 5)**. **(A) **FLSs were cultured in the presence of TNF-α (20 ng/ml) with or without atorvastatin (50 to 100 μ*M*) for 24 hours to determine the effect of atorvastatin on the activations of signaling molecules. Phosphorylated ERK, JNK, Akt, and p38 MARK were analyzed by using Western blotting. Representative Western blots from three independent experiments are shown. **(B) **The effect of atorvastatin on the phosphorylation of p38 MARK induced by TNF-α in FLSs from an RA patient. After serum deprivation for 24 hours, FLSs were cultured with SB203580 (10 μ*M*) for 2 hours. TNF-α (20 ng/ml) and atorvastatin (50 to 100 μ*M*) were then added, and incubation was continued for another 24 hours. The expressions of RANKL mRNA relative to GAPDH were determined with RT-PCR. **(C) **RANKL secretion was determined with ELISA. Both SB203580 and atorvastatin inhibited RANKL production by TNF-α.

To investigate the mechanism by which atorvastatin suppresses the expression of TNF-α-induced RANKL, we examined its effects on the phosphorylations of p38 MAPK, JNK, ERK, and Akt. Western blotting revealed that atorvastatin inhibited TNF-α-induced p38 phosphorylation after 12 hours, but that it did not affect the phosphorylations of JNK, ERK, and Akt (Figure 5A). When we pretreated cells with SB203580 (a p38 inhibitor), TNF-α-induced RANKL mRNA expression was decreased, which was similar to the suppression of RANKL mRNA expression by atorvastatin (Figure 5B). We also measured soluble RANKL levels in FLSs culture media, and we found that its levels were decreased by SB203580 and by atorvastatin (Figure 5C).

### Inhibition of osteoclast formation

**Figure 6:**
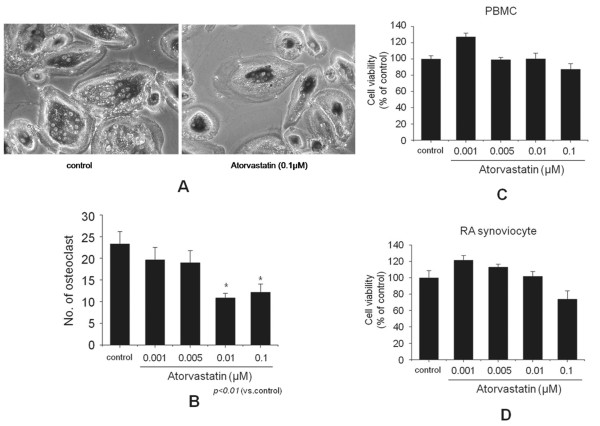
**Effect of atorvastatin on osteoclast formation**. **(A) **Osteoclast formation was assayed with tartrate-resistant acid phosphatase (TRAP) staining and counting positive cells containing three or more nuclei under a light microscope (original magnification, ×100). **(B) **Peripheral blood mononuclear cells (PBMCs; 2 × 10^5 ^cells/well) from a healthy donor were cocultured with fibroblast-like synoviocytes from a rheumatoid arthritis patient (RA FLSs; 2 × 10^4 ^cells/well, patients 1, 4, and 5) in the presence of macrophage colony-stimulating factor, 1,25-dihydroxyvitamin D_3_, and different concentrations (0.001 to 0.1 μ*M*) of atorvastatin in 96-well plates for 3 weeks. Cell viabilities were assayed in separate cultures of FLSs and PBMCs. **(C) **PBMCs were cultured for 3 weeks in the presence of 10^-7 ^*M *1,25-dihydroxyvitamin D_3 _and different concentrations of atorvastatin. **(D) **RA FLSs were cultured for 3 weeks in the presence of 2 ng/ml macrophage colony-stimulating factor and different concentrations of atorvastatin (0.001 to 0.1 μ*M*). Bars represent means and SDs. All experiments were carried out 3 times in triplicate. *P < 0.01 versus control.

It was reported that RANKL is produced by RA FLS. We added 1,25-dihydroxyvitamin D_3 _and M-CSF to media in which FLSs or cocultured cells were grown, according to a previously described method [3]. We could produce functionally active osteoclasts, and they were determined by TRAP staining (Figure 6A). When atorvastatin was added to cocultures of PBMCs and FLSs for 3 weeks, atorvastatin at ≥0.01 μ*M *significantly decreased the numbers of TRAP-positive multinucleated osteoclasts (*P *< 0.01 versus control) (Figure 6A, B). To ensure that the inhibitory effect of atorvastatin on osteoclast formation was not due to an effect on cell viability, we performed MTT assay. Cell viabilities were assayed in separate cultures of FLSs and PBMCs. The viability of PBMCs after 3 weeks of treatment in the presence of M-CSF was not affected by atorvastatin (Figure 6C). Furthermore, atorvastatin had no effect on FLS viability after 3 weeks of treatment in the presence of vitamin D_3 _(Figure 6D).

## Discussion

Statins inhibit the rate-limiting step of cholesterol synthesis by preventing HMG-CoA from being reduced to mevalonate by HMG-CoA reductase [21]. In addition, statins appear to have beneficial therapeutic effects on several human diseases, such as multiple sclerosis (MS) [22] and osteoporosis [23], which have no direct association with cholesterol levels. Recently, a number of reports have been issued on the antiinflammatory potentials of statins in rheumatic disease [18,19,24,25] Therefore, we hypothesized that atorvastatin could have beneficial effects on the progressive joint destruction in RA.

In a preliminary study, we confirmed that TNF-α induced RANKL in FLSs from RA patients after incubation for 24 hours, which is consistent with that found by Kubota *et al*. [26]. In the present study, we found that atorvastatin inhibited the expression of TNF-α-induced RANKL in FLSs from RA patients. Simvastatin also significantly and dose-dependently inhibited the expression of RANKL mRNA and protein in the FLSs of RA patients. Mevalonate not only is a substrate for cholesterol biosynthesis, but also is required for the synthesis of several other biologically important lipid intermediates by means of an alternative synthesis pathway [27]. Furthermore, it is well known that mevalonate prevents the effects of statins, and in the present study, we found that the suppression of RANKL expression by statins is prevented by mevalonate. Osteoclasts play an important role in rheumatoid bone erosion in RA [28-30], and RANKL plays an important role in differentiation of osteoclasts from their precursor cells and promotes the activity and survival of these cells, which leads to bone resorption [31]. In the present study, the results were obtained by using a coculture system for osteoclastogenesis and showed that atorvastatin inhibits osteoclast formation. To our knowledge, this is the first report describing that atorvastatin inhibits osteoclastogenesis by attenuating RANKL expression in the FLSs of RA.

In the present study, we performed cocultures at lower atorvastatin concentrations (0.001 to 0.1 μ*M*). Exposure to higher concentrations (1 μ*M *or more) for 3 weeks was found to be toxic to both FLSs and PBMCs (data not shown).

The receptor activator of the NF-κB (RANK)/RANKL system has been shown to be a critical factor during osteoclast differentiation and bone resorption [31-33]. In addition, osteoprotegerin (OPG) functions as a high-affinity soluble decoy receptor for RANKL and competes with RANK for RANKL binding. Therefore, OPG is an effective inhibitor of the RANK-RANKL interaction. Several research groups have assessed the role of the RANK/RANKL/OPG system in RA, and experimental arthritis models have been established to study the *in vivo *effects of these molecules [4]. Recently, RANKL was reported to participate in osteoclast formation in a model of spontaneously developing erosive arthritis in the mouse [30]. However, in the present study, atorvastatin did not enhance the expressions of OPG protein and mRNA, and atorvastatin did not affect RANK expression in CD14^+ ^cells. These findings indicate that the inhibition of osteoclast formation by atorvastatin is independent of OPG and RANK.

Signaling pathways that regulate proinflammatory mediator expression in FLSs of RA patients include MAPKs and NF-κB. Three MAPK families have been implicated in RA: ERK1/2, JNK, and p38 MAPK [34]. Interestingly, all three of these MAPK families are activated in RA synovial tissue and in cultured FLSs from RA patients. Furthermore, TNF-α has the potential to signal through all three [35]. However, we observed that atorvastatin decreased the phosphorylation of p38 but not that of ERK 1/2 or JNK, and that SB203580 (a specific p38 inhibitor) had a similar inhibitory effect on RANKL production in the FLSs of RA patients. Therefore, p38 may play a central role in which atorvastatin suppresses the expression of RANKL by TNF-α.

## Conclusions

The present study shows that atorvastatin markedly reduce RANKL expression in the fibroblast-like synoviocytes of rheumatoid arthritis patients, and atorvastatin inhibits osteoclastogenesis in co-cultures of PBMCs and FLSs. These results suggest that atorvastatin may inhibit osteoclastogenesis and bone destruction in RA patients.

## Abbreviations

DMEM-HG: Dulbecco Modified Eagle Medium-High Glucose; dNTP: deoxynucleotide triphosphate; DTT: dithiothreitol; ELISA: enzyme-linked immunosorbent assay; FLS: fibroblast-like synoviocyte; GAPDH: glyceraldehyde 3-phosphate dehydrogenase; HMG-CoA: hydroxymethylglutaryl-coenzyme A; MAPK: mitogen-activated protein kinase; M-CSF: macrophage colony-stimulating factor; MTT: methylthiazol tetrazolium; OPG: osteoprotegerin; PBMC: peripheral blood mononuclear cell; PBS: phosphate-buffered saline; PI staining: propidium iodide staining; RA: rheumatoid arthritis; RANK: receptor activator of nuclear factor; RANKL: receptor activator of nuclear factor κB ligand; RT-PCR: reverse transcription-polymerase chain reaction; TNF-α: tumor necrosis factor α; TRAP: tartrate-resistant acid phosphatase.

## Competing interests

The authors declare that they have no competing interests.

## Authors' contributions

YWS was responsible for designing the study, access to samples, and manuscript preparation. JYK designed the study and performed most of the experiments, data analysis, and wrote the manuscript. EYL was responsible for designing the study and osteoclast experiments. EBL, YJL, and HJY contributed to the conception of the study and interpretation of the data. JYC performed qRT-PCR. All authors read and approved the final manuscript.

## Supplementary Material

Additional file 1 **Figure S1. Effects of simvastatin on receptor activator of nuclear factor κB ligand (RANKL) expression in fibroblast-like synoviocytes (FLSs) from rheumatoid arthritis (RA) patients (patients 1, 2, and 3)**. FLSs were isolated from three RA patients and cultured in the presence of TNF-α (20 ng/ml) with or without simvastatin (10 to 100 μ*M*) for 24 hours. RANKL expressions were analyzed with RT-PCR **(A) **and Western blotting **(B)**. Click here for file

Additional file 2 **Figure S2. Effects of mevalonate on the statin-induced suppression of RANKL**. FLSs from RA patients (patients 1, 2, and 3) were cultured in the presence of TNF-α (20 ng/ml), and then simvastatin (50 to 100 μ*M*) and mevalonate (100 μ*M*) were added for 24 hours. RANKL expressions were analyzed with RT-PCR **(A) **and Western blotting **(B)**. Click here for file

Additional file 3 **Figure S3. The effects of simvastatin on cell viability and apoptosis**. **(A) **Effects of simvastatin on the viability of FLSs from an RA patient (patients 1, 2, and 3). Cells were cultured for 24 hours in the presence of TNF-α (20 ng/ml) and simvastatin at different concentrations (10 to 100 μ*M*). Cell viabilities were determined by using MTT assays. **(B) **Effects of simvastatin on the apoptosis of FLSs, as determined by propidium iodide staining. Click here for file
