# Supplementary figures and images for: Atorvastatin inhibits osteoclastogenesis by decreasing the expression of RANKL in the synoviocytes of rheumatoid arthritis
Source: Arthritis Res Ther. 2012 Aug 17;14(4):R187. doi: 10.1186/ar4018 (PMC3580583; doi:10.1186/ar4018)

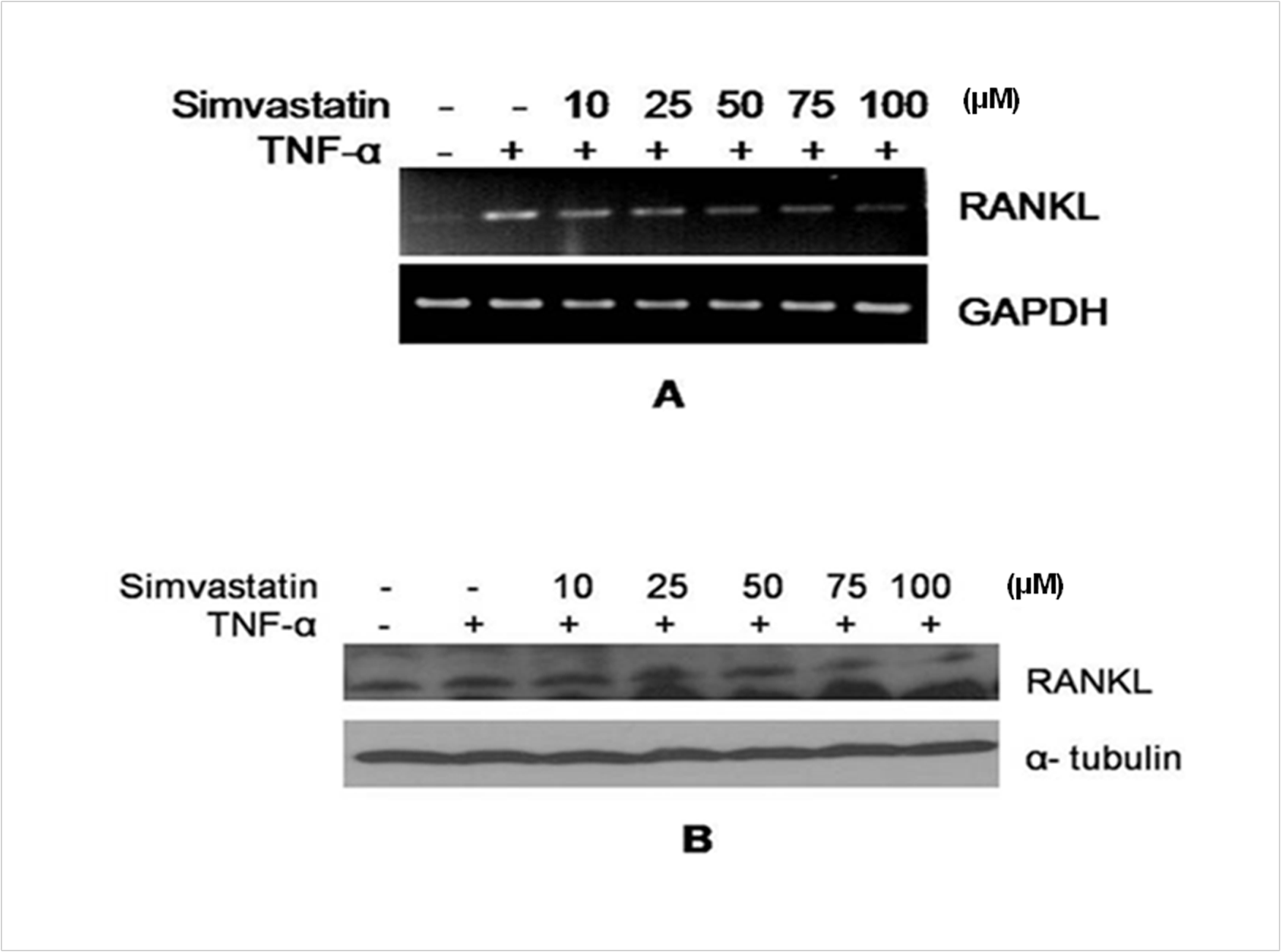

Supplement: Additional file 1 — Figure S1. Effects of simvastatin on receptor activator of nuclear factor κB ligand (RANKL) expression in fibroblast-like synoviocytes (FLSs) from rheumatoid arthritis (RA) patients (patients 1, 2, and 3). FLSs were isolated from three RA patients and cultured in the presence of TNF-α (20 ng/ml) with or without simvastatin (10 to 100 μM) for 24 hours. RANKL expressions were analyzed with RT-PCR (A) and Western blotting (B). [file ar4018-S1.TIFF]

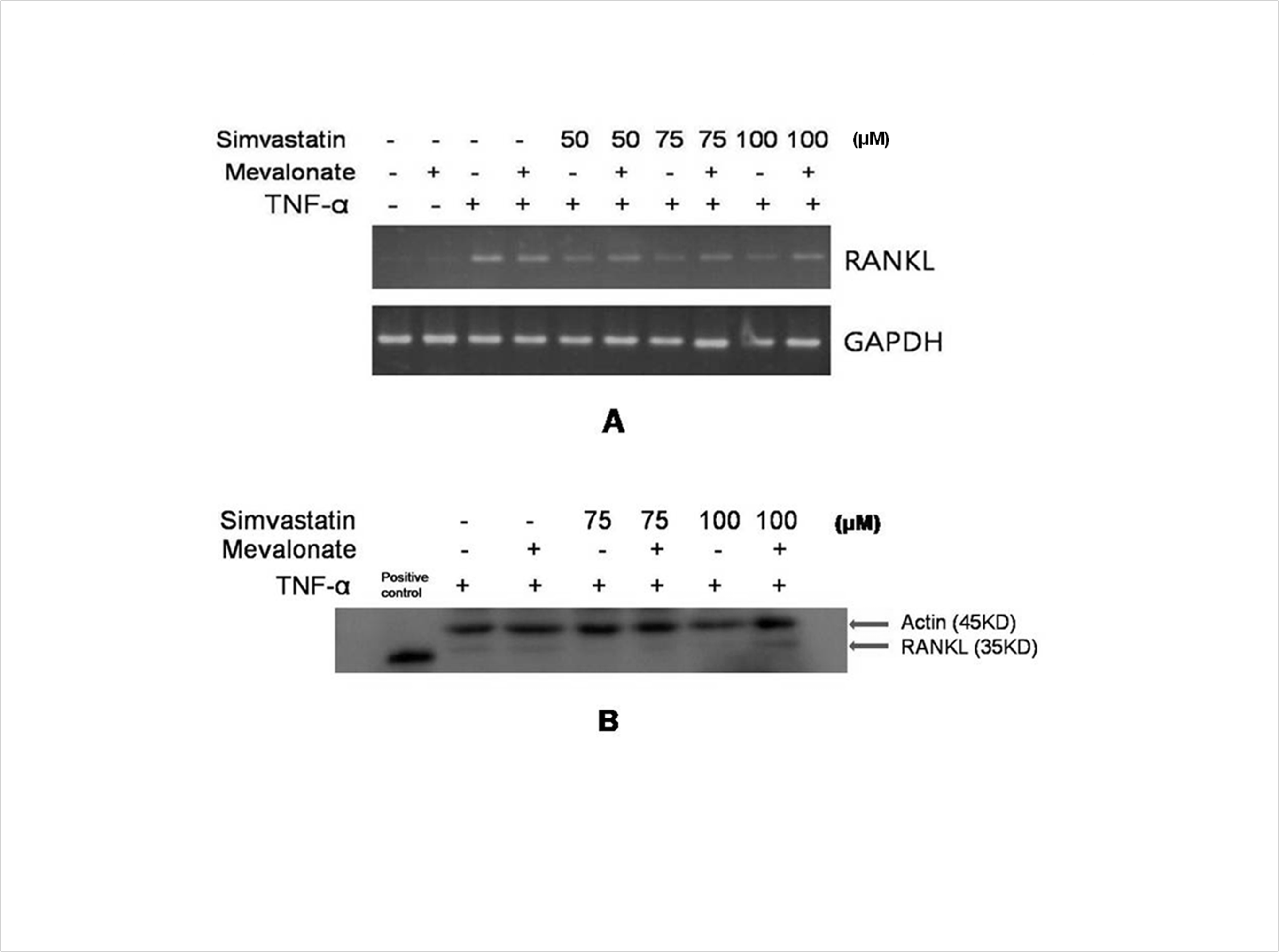

Supplement: Additional file 2 — Figure S2. Effects of mevalonate on the statin-induced suppression of RANKL. FLSs from RA patients (patients 1, 2, and 3) were cultured in the presence of TNF-α (20 ng/ml), and then simvastatin (50 to 100 μM) and mevalonate (100 μM) were added for 24 hours. RANKL expressions were analyzed with RT-PCR (A) and Western blotting (B). [file ar4018-S2.TIFF]

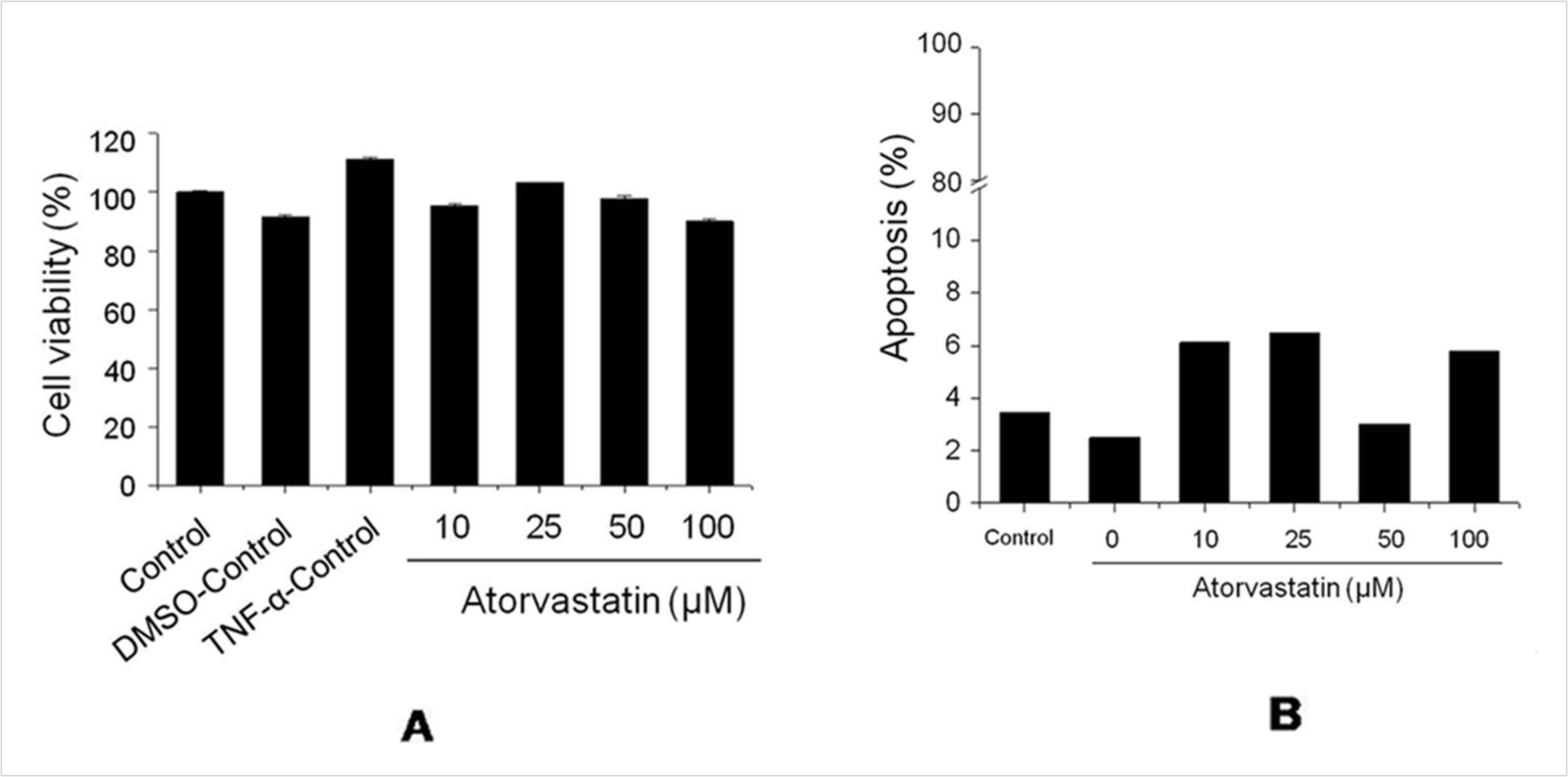

Supplement: Additional file 3 — Figure S3. The effects of simvastatin on cell viability and apoptosis. (A) Effects of simvastatin on the viability of FLSs from an RA patient (patients 1, 2, and 3). Cells were cultured for 24 hours in the presence of TNF-α (20 ng/ml) and simvastatin at different concentrations (10 to 100 μM). Cell viabilities were determined by using MTT assays. (B) Effects of simvastatin on the apoptosis of FLSs, as determined by propidium iodide staining. [file ar4018-S3.TIFF]
